# Direct and Indirect Links Between Peer Relationships, Father Involvement and Self-Harm Among Rural Chinese Adolescents

**DOI:** 10.3390/children13101426

**Published:** 2026-10-17

**Authors:** Minfei Yu, Stephen Houghton, Elaine Chapman

**Affiliations:** Graduate School of Education, The University of Western Australia, Perth, WA 6009, Australia; minfei.yu@research.uwa.edu.au (M.Y.); stephen.houghton@uwa.edu.au (S.H.)

**Keywords:** self-harm, depression, anxiety, peer relationships, father involvement, rural adolescents, structural equation modelling

## Abstract

**Highlights:**

**What are the main findings?**

- Among 588 rural adolescents aged 12–16 years, depression showed the strongest association with self-harm (*β* = 0.37), whereas anxiety was only marginally related (*β* = 0.12, *p* = 0.06). Both peer relationships and father involvement were linked to self-harm mainly through depression.
- The total association for peer relationships (*β* = −0.23) was larger than that of father involvement (*β* = −0.16). Neither direct path to self-harm was significant; the protective associations operated mainly through the indirect depression pathway.

**What are the implications of the main findings?**

- Peer relationships showed a stronger protective association with self-harm than father involvement, and depression was the main pathway for both. The results can guide assessment, prevention and intervention in rural schools, including depressive symptom screening and peer support. Father involvement was also protective, but the association was smaller.
- Rural areas in China have fewer resources, and in low-income households, fathers often migrate for work, so left-behind children receive less day-to-day care from their fathers. The smaller father involvement association in this sample agrees with that pattern. Peer support and school-based support can be strengthened together with father involvement.

**Abstract:**

**Objectives:** Epidemiological surveys in rural China have reported comparatively high adolescent self-harm rates. Evidence on how peer relationships and father involvement jointly relate to this risk and on whether depression or anxiety helps to explain those links is more limited. This study investigated peer relationships and father involvement as two potential protective correlates of self-harm and assessed whether associations with self-harm were linked via depression and anxiety in a cross-sectional model. **Methods:** This study analysed publicly available data from the China Rural Children Mental Health Database. Data were collected in 16 rural primary schools across seven provinces. A sample of 588 adolescents aged 12–16 years with complete key variables was included. Structural equation modelling examined indirect associations between peer relationships, father involvement and self-harm through depression and anxiety. **Results:** Depression was positively and significantly associated with self-harm (*β* = 0.37, *p* < 0.001), while anxiety demonstrated only a marginally significant association with self-harm (*β* = 0.12, *p* = 0.06). Both peer relationships and father involvement were significantly associated with lower depression levels. The total associations for peer relationships (−0.23) exceeded those for father involvement (−0.16). The indirect associations of both variables with self-harm were linked primarily via depression. **Conclusions:** In this cross-sectional model, depression was the main pathway linking peer relationships and father involvement with self-harm, and the association for peer relationships was stronger than that for father involvement. Our results can guide assessment, prevention and intervention among adolescents in rural Chinese schools, including peer support and screening for depressive symptoms.

## 1. Introduction

### 1.1. Self-Harm and the Rural Chinese Context

Self-harm refers to deliberate acts of bodily injury occurring with or without suicidal intent [1]. Non-suicidal self-injury (NSSI) is a narrower term that refers only to self-injury without suicidal intent, such as cutting, burning, hitting or biting [2,3]. The present study focuses on deliberate self-harm as a broad behavioural outcome. Even so, self-harm remains closely linked to later suicide risk: young people with a history of self-injurious behaviour are substantially more likely to report suicidal ideation and subsequent suicide attempts [4]. Adolescent self-harm is included in this study as an early indicator of later suicide risk. Over the past decade, adolescent self-harm has become a major global public health concern, and rates appear to have risen in several countries; for example, longitudinal research in Norway found about a fourfold increase over 15 years [5].

Among 22 health problem categories reported for young people in China, mental disorders have become the leading cause of health-adjusted life years lost among children and adolescents [6]. School studies of Chinese students have also reported depressive symptoms and anxiety symptoms. Chen et al. [7] reported detection rates of 23.0% for depressive symptoms and 13.9% for anxiety symptoms among secondary school students in Zigong, Western China. High rates of adolescent self-injurious behaviour have also been reported in Chinese primary, middle and high school samples [8].

In rural China, adolescent self-harm risk appears elevated. For example, Tang et al. [9] reported that approximately 29% of rural adolescents had engaged in at least one act of self-harm in the past year, and rural female adolescents have shown even higher rates [10]. The risks and protections faced by rural adolescents in China are not the same as those in city samples. Various factors might contribute to the increased risk rates seen in rural populations. For example, parent–child separation resulting from paternal labour migration is more prevalent in rural than in metropolitan areas. Paternal absence diminishes opportunities for fathers to provide emotional care and cognitive guidance to their children [11,12]. Population mobility in rural areas may also contribute to poor peer network stability in young people. Peer relationship disruptions may undermine important mental health protections such as social support, putting this group more at risk [9,13]. Rural areas tend also to have limited mental health service resources, which makes early intervention for mental health problems less likely [14].

### 1.2. External and Internal Factors

For rural adolescents, peer relationships and father involvement are two relationships outside the individual. Self-harm often persists or recurs after onset [15], and depression, distress and adverse family or social relationships are associated with both initial and repeated self-harm [16]. Mental health services and referral in rural China are often limited, so peer relationships and father involvement are among the few things that schools there can still observe.

Theoretically, the factors that influence adolescent self-harm can be distinguished into internal and external factors. External factors primarily refer to social relationships and situational conditions that are relevant to the individual, such as family care and functioning, school environments, availability of social support, life stressors and broader socioeconomic backgrounds [17]. These factors are important external contexts for self-harm risk. External factors such as high levels of mobility can serve to increase self-harm risk, whereas factors such as cohesive family structures and positive peer relationships can serve as protective factors that decrease longer-term self-harm risks.

Internal factors primarily refer to emotional, cognitive and regulatory processes within individuals, such as resilience, coping strategies, depression, anxiety, hopelessness, self-criticism and emotion regulation difficulties [18]. These factors are generally more proximal in time to self-harm events and often mediate links between external factors and adverse mental health events. For example, internal factors can determine whether individuals will resort to self-harm behaviours when confronted with external stressors such as changing schools [1,4]. External conditions and these internal processes are both part of the account of self-harm risk used here.

Parent and peer relationships are key contexts for adolescent mental health, and difficulties in these relationships—such as poor attachment, harsh punishment, bullying or low social support—are closely related to depression, anxiety and self-harm [19,20,21,22,23].

### 1.3. Peer Relationships and Adolescent Self-Harm

Peer relationships represent a double-edged sword in adolescent development. On the one hand, positive peer support can serve as an important protective factor, buffering the negative effects of family adversity on mental health [24]. In this process, peers provide not only emotional responses but may also influence adolescent emotional trajectories and coping choices through peer approval and daily interactions, thereby reducing risks from accumulated persistent negative emotions [25]. Poor peer relationships may also become risk sources. Lack of peer support may be associated with increased loneliness and inner distress [22,26].

When adolescents lack sufficient emotion regulation skills, they may be more inclined to adopt self-harm behaviours as coping strategies [27]. Negative peer experiences such as peer bullying, rejection or persistent peer pressure reinforce adolescent self-negation and self-criticism, forming associations with self-harm occurrence by exacerbating internalising symptoms such as depression [28].

### 1.4. Father Involvement and Adolescent Self-Harm

Parent–child relationships and parental involvement are also considered important foundations for adolescent emotional development and coping styles. Previous research indicates that high-quality family relationships (such as parent–child trust and effective communication) are associated with lower levels of antisocial behaviour as well as lower anxiety, depression and loneliness levels in adolescents [29]. These positive psychological states may prompt adolescents to maintain more stable emotional levels [30] and to adopt more positive coping approaches and help-seeking behaviours. Collectively, these have been found to reduce the likelihood of self-harm behaviours from developing [31]. Higher levels of parent–child alienation may increase the risk of self-harm behaviours developing [31]. For example, Victor et al. [32] found through studies of longitudinal cohorts that harsh parental punishment, low parental monitoring, and poor parent–child attachments predict subsequent NSSI occurrence among adolescents.

Adolescence brings rapid psychological and physiological change, and when adolescents cannot process negative experiences in family relationships, they may use self-harm to relieve distress [32]. However, research results regarding mother–child relationships (MCRs) and father–child relationships (FCRs) similarly demonstrate heterogeneity. Traditional attachment theory considers mothers more typical secure attachment figures, while fathers are more likely play roles in facilitating children’s exploratory systems [33]. Over time, fathers have increasingly been recognised to play a vital role in family caregiving and child-rearing [34,35].

Father involvement is defined as the emotional, cognitive and behavioural guidance and investment fathers provide to enable children’s healthy development [34]. It encompasses three dimensions: accessibility, engagement and responsibility [35]. Research indicates that young people with healthy father–child relationships show fewer problem behaviours in both the internalising and the externalising domains [10]. In particular, increased father involvement has been found to be associated with reduced emotional burdens, decreased negative emotion levels, and more stable psychological adaptation processes among adolescents [10]. Peer relationships and father involvement were therefore examined separately in the same model.

### 1.5. Depression and Anxiety as Pathways

Depression and anxiety are closely related to adolescent self-harm [4,36]. In Nock and Prinstein’s [37] functional model, self-injury is linked with difficulty in managing strong negative feelings. Depression and anxiety were therefore placed on the paths from the social variables to self-harm. In Cohen and Wills’ [38] buffering account, more supportive relationships are associated with lower distress. Peer relationships and father involvement were the two sources of support in the model, with paths running from each of these variables to depression and to anxiety and from depression and anxiety to self-harm.

Reviews also suggest that self-harm is usually tied more directly to depression-related processes, whereas links between anxiety and self-harm often depend on comorbidity with depression or on more specific difficulties [39]. Symptom network research in rural China likewise points to depressive symptoms as a bridge between anxiety and self-harm [40]. Depression and anxiety were therefore modelled as parallel mediators so that their indirect associations could be compared. Much of this theoretical work comes from research on NSSI. It is drawn on here in considering pathways to self-harm. The outcome was a broad self-harm question and did not separate suicidal intent. Experiential avoidance accounts similarly suggest that self-harm may be maintained through short-term psychological relief [1,41].

### 1.6. The Present Study

While peer relationships, father involvement, depression and anxiety have each been examined in relation to adolescent self-harm, fewer studies have tested these social protective factors together in one model or compared their relative associations with self-harm through depression and anxiety. Existing evidence also comes mainly from urban or general population samples [5,24,42,43]. Research focusing specifically on rural Chinese adolescents, who face comparatively high self-harm risk, remains more limited.

The present study therefore used structural equation modelling to examine whether peer relationships and father involvement were significantly associated with self-harm among rural adolescents in China. Depression and anxiety were also examined as potential indirect pathways in these associations. This study addresses two primary research questions:

1. Among rural adolescents in China, are peer relationships and father involvement significantly associated with reported self-harm behaviours?
2. In this cross-sectional model, are the associations of peer relationships and father involvement with self-harm accounted for by anxiety or by depression?

## 2. Materials and Methods

### 2.1. Participants

This study used publicly available data from the “China Rural Children Public Mental Health Database,” representing secondary analysis. The original data collection was funded by the Chinese Academy of Sciences Informatization Plan (CAS-WX2021PY-0204) and the Chinese Academy of Sciences Special Research Assistant Project (E2CX0114), with corresponding ethical approval obtained. Data were collected from March to May 2021 at 16 rural primary schools across 7 provinces: Anhui, Gansu, Guangdong, Heilongjiang, Hubei, Hunan and Sichuan. All participants and their guardians provided written informed consent, and all responses were collected anonymously. Because this study only used anonymised open data without direct participant contact, a waiver of ethical review was granted by the University of Western Australia Human Ethics Committee. Based on the research objectives for the present study, we extracted participants who completed the Children’s Depression Inventory (CDI-27), the Generalized Anxiety Disorder Scale (GAD-7), the Father Involvement Questionnaire (FIQ), the Peer Relationships Scale (PR) and the single self-harm item from the original database. The primary analysis sample was limited to adolescents aged 12–16 years (*N* = 588).

### 2.2. Research Model and Hypothesis Formulation

**Figure 1:**
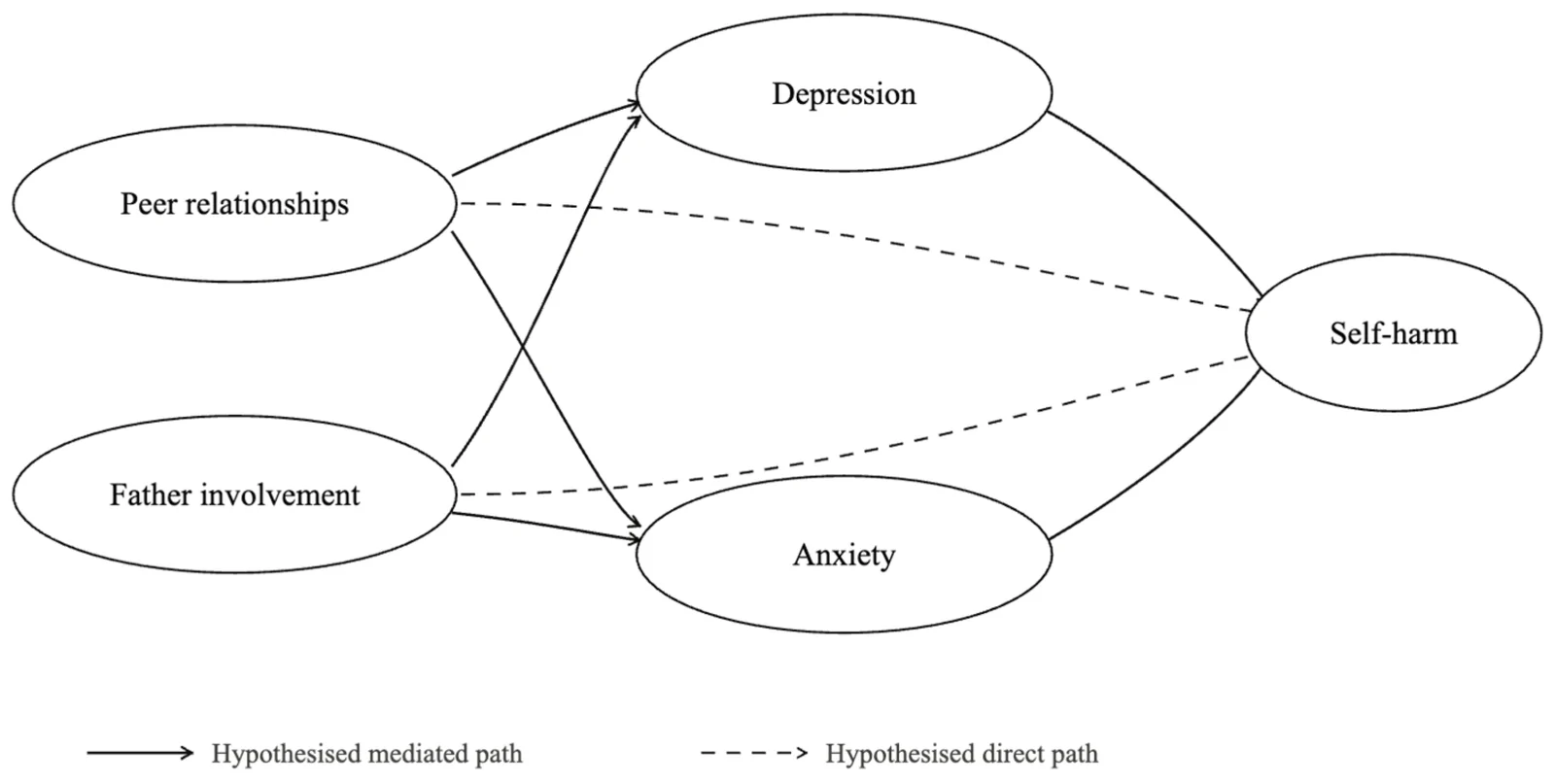
Hypothesised structural model linking peer relationships and father involvement to self-harm, with possible mediating links via depression and anxiety.

The model was specified from two theoretical accounts. In Nock and Prinstein’s [37] functional model, self-harm is one way of handling strong negative feelings. Depression and anxiety were therefore set as parallel mediators between the social variables and self-harm. In Cohen and Wills’ [38] buffering account, social support is associated with lower distress. Peer relationships and father involvement were therefore the exogenous supports. Direct paths from peer relationships and father involvement to self-harm were also estimated (see Figure 1).

### 2.3. Measures

#### 2.3.1. Depressive Symptoms

The Chinese version of the 27-item Children’s Depression Inventory (CDI-27) assessed depressive symptoms in children aged 6–17 years over the past two weeks. The scale consists of 27 items scored on a 3-point scale, with each item comprising sentences describing varying degrees of depressive symptoms: 0 = no symptoms, 1 = mild symptoms, 2 = definite symptoms. Total scores range from 0 to 54, with higher scores indicating greater depression. The Chinese CDI-27 has been found to demonstrate high levels of reliability and validity in Chinese child and adolescent populations [44].

#### 2.3.2. Anxiety Symptoms

The Chinese version of the 7-item Generalized Anxiety Disorder Scale (GAD-7) measured anxiety symptoms over the past two weeks. The scale comprises 7 items scored on a 4-point scale (0 = not at all, 1 = several days, 2 = more than half the days, 3 = nearly every day), with total scores ranging from 0 to 21. Higher scores indicate higher anxiety levels. Common severity cutoffs are mild ≥ 5, moderate ≥ 10, severe ≥ 15 [45]. The Chinese GAD-7 has been found to exhibit sound reliability and validity properties in Chinese populations, including adolescents [46].

#### 2.3.3. Father Involvement

The father involvement scale was adapted from Wu et al.’s [47] Father Involvement Questionnaire (FIQ), culturally adapted for Chinese family contexts. This resulted in a 22-item Chinese version. The adapted scale assesses children’s perceived father involvement across dimensions including daily care, academic support and emotional communication. Items are scored on a 5-point Likert scale from 0 (never) to 4 (always), with total scores ranging from 0 to 88. Higher scores indicate greater father involvement. Again, this adapted scale has demonstrated high levels of reliability and validity among Chinese children and adolescents [47].

#### 2.3.4. Peer Relationships

Peer relationships were measured with the eight-item Chinese brief peer relationship scale used in the source survey [48]. Each item was scored from 0 (never) to 4 (always). The items were summed (range 0–32), with a higher score indicating better peer relationships. Fang et al. [48] describe this brief scale as the Chinese form of the PROMIS Pediatric Peer Relationships measure [49]. In that survey, the internal consistency was 0.86.

#### 2.3.5. Deliberate Self-Harm

Deliberate self-harm was measured using a single self-report item. Participants answered “Have you ever deliberately hurt yourself?” with response options of yes/no, reflecting lifetime self-harm prevalence, coded as a binary variable (0 = no, 1 = yes). In large school surveys, a single item is often used to record whether self-harm has occurred [50].

The item does not record when the self-harm occurred, how often it occurred, how serious it was, which method was used, or whether suicidal intent was present. A “yes” may be one act a long time ago or repeated self-harm more recently. The item is a lifetime screen, not a diagnosis of NSSI.

### 2.4. Statistical Methods

All analyses were conducted using R 4.5.1 [51], with structural equation modelling implemented using the lavaan package (version 0.6-21) [52]. Tests were two-tailed, with statistical significance set at *p* < 0.05. Descriptive statistics summarised the sample, including the proportion who reported self-harm and the means and standard deviations of depression, anxiety, peer relationships and father involvement. The main analysis was a structural equation model with peer relationships and father involvement as exogenous variables, depression and anxiety as parallel mediators, and self-harm as a binary outcome.

Because the outcome was binary, an observed-variable path model was estimated with WLSMV and a probit link. Peer relationships, father involvement, depression and anxiety were entered as scale total scores. Standardised path coefficients (*β*), robust standard errors, 95% confidence intervals and *p*-values were reported, and indirect associations were estimated with the product-of-coefficients method. Scale totals were used rather than latent factors, because the aim was to estimate paths among these measures rather than to develop a new measurement model. Self-harm was a single yes/no item and could not be treated as a latent outcome with several indicators. All structural paths were freely estimated. Under this specification, the single-group model is saturated (df = 0). Global fit indices would not add information and were not interpreted. The reported results are the path coefficients, the indirect associations, and R^2^.

Age (in years), sex (0 = male, 1 = female), and left-behind status (0 = not left behind, 1 = father or both parents had migrated for work) were entered as covariates in the equations for depression, anxiety and self-harm. Peer relationships and father involvement remained exogenous predictors. Covariances between these predictors and the three covariates and among the covariates were freely estimated, so age, sex and left-behind status were allowed to correlate with the exogenous variables. No directed paths were specified from the covariates to peer relationships or father involvement. Adjustment was carried by the covariate paths to depression, anxiety and self-harm, together with those free covariances. The model was also estimated separately by sex. The main paths were similar for boys and girls in direction and magnitude, so the full-sample results are reported.

The same structural model was estimated again without the covariates, so that the adjusted and unadjusted main paths could be compared. A further model omitted depression and retained anxiety as the only mediator.

## 3. Results

Prior to model estimation, this study completed data screening and tested path analysis assumptions. Results indicated that relationships among main variables generally conformed to linear specifications; variable and residual distributions were acceptable; error terms showed no systematic correlations, and no correlated residuals were specified; and no multicollinearity was observed among the predictor variables. Overall, the screening analyses performed affirmed that data satisfied all assumptions underlying the use of path analysis. To test the stability of the proposed pathway structure across gender, a multi-group analysis was initially conducted separately for male and female adolescents. This analysis indicated that the direction and magnitude of all main structural paths were consistent across groups. In light of this, only the full-sample results are presented here.

### 3.1. Sample Characteristics and Group Comparisons

The primary analysis sample comprised 588 rural adolescents aged 12–16 years (321 females, 54.6%; 267 males, 45.4%). Overall, 156 adolescents (26.5%) reported at least one self-harm act. Females reported having self-harmed more often than did males (32.4% vs. 19.5%). After further stratification by left-behind status, left-behind females showed the highest self-harm rates (37.3%), followed by non-left-behind females (27.6%), left-behind males (20.0%) and non-left-behind males (19.0%). Table 1 presents mean comparisons on key variables between adolescents with and without self-harm. Adolescents with self-harm behaviour scored significantly higher on depression (*M* = 20.38 vs. 12.69, *d* = 1.04, *p* < 0.001) and anxiety (*M* = 8.74 vs. 5.11, *d* = 0.79, *p* < 0.001) and significantly lower on peer relationships (*M* = 14.63 vs. 18.12, *d* = −0.51, *p* < 0.001) and father involvement (*M* = 31.31 vs. 40.47, *d* = −0.46, *p* < 0.001) (see Table 1).

### 3.2. Hypothesis Testing and Association Estimates

The primary path model was based on 588 adolescents aged 12–16 years with complete key variables. This model is saturated when estimated in a single group (df = 0); therefore, global fit indices are not emphasised in interpretation. The focus is instead on the obtained path coefficients, indirect associations and variance explained. The model explained 31%, 11% and 31% of variance in the depression, anxiety and self-harm variables, respectively. Depression was positively and significantly associated with self-harm (*β* = 0.37, *p* < 0.001), while the relationship between anxiety and self-harm was only marginally significant (*β* = 0.12, *p* = 0.06). This result suggests that in this sample, depression accounted for more of the association between the external variables and self-harm than anxiety did.

**Figure 2:**
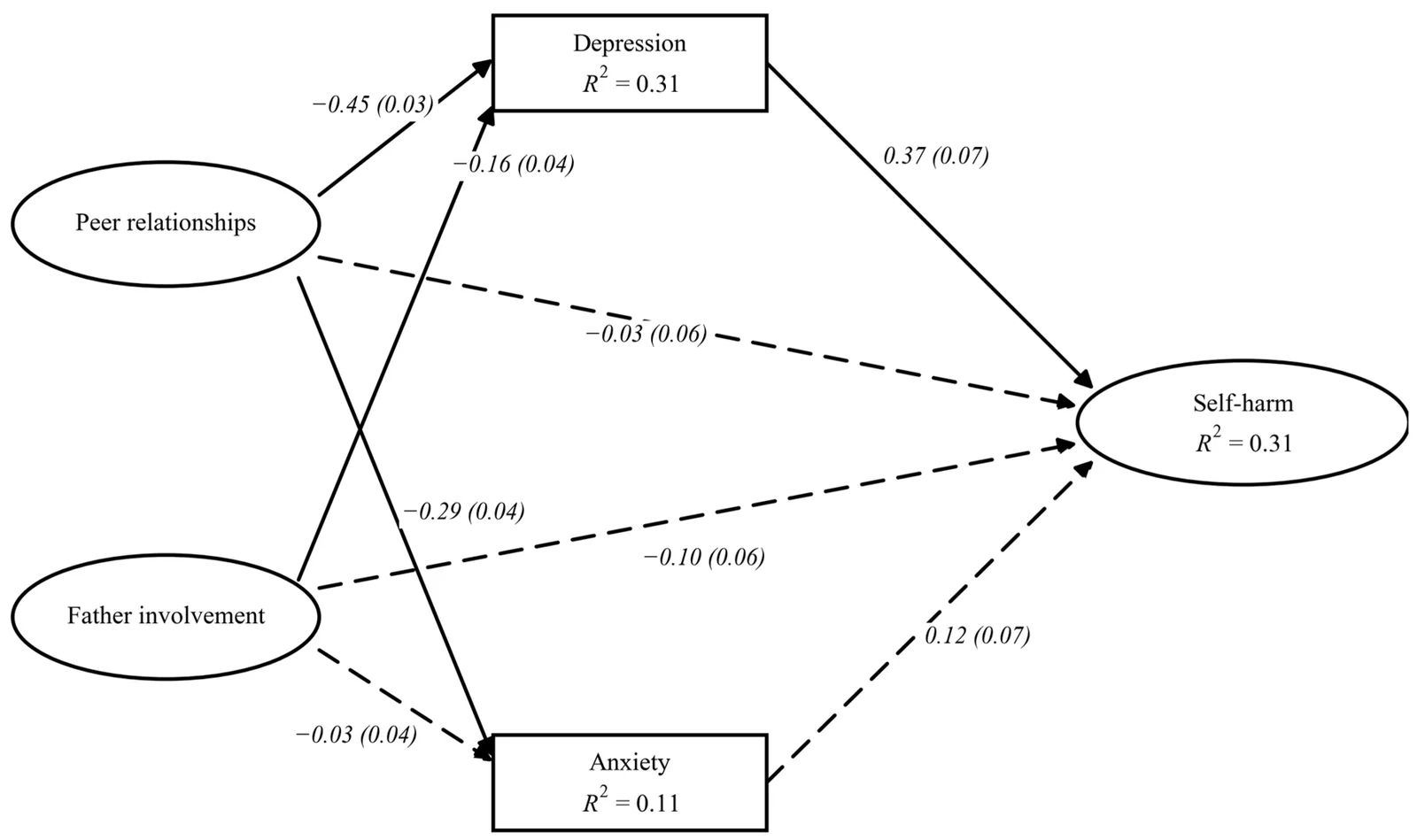
Structural equation model linking peer relationships and father involvement with self-harm via depression and anxiety. Values are standardised path coefficients with robust standard errors in parentheses. Solid arrows indicate paths significant at *p* < 0.05; dashed arrows indicate non-significant paths. Age, sex and left-behind status were included as covariates in the equations for depression, anxiety and self-harm but are omitted from the figure for clarity.

Peer relationships showed significant negative associations with both depression (*β* = −0.45, *p* < 0.001) and anxiety (*β* = −0.29, *p* < 0.001). Father involvement also showed a significant negative association with depression (*β* = −0.16, *p* < 0.001) but not with anxiety (*β* = −0.03, *p* = 0.42) (see Table 2). The direct path from peer relationships to self-harm was non-significant (*β* = −0.03, *p* = 0.66), as was the direct path from father involvement (*β* = −0.10, *p* = 0.09). However, the indirect association of peer relationships with self-harm through depression was significant (*β* = −0.17, *p* < 0.001), as was the indirect association of father involvement with self-harm through depression (*β* = −0.06, *p* < 0.01). Neither indirect association through anxiety was significant. The total association for peer relationships was larger than that for father involvement (peer: *β* = −0.23, *p* < 0.001; father: *β* = −0.16, *p* = 0.01). These results indicate that peer relationships showed the stronger protective association in this model. Father involvement also showed a significant, albeit weaker, indirect protective association, with both associations linked primarily via the depression pathway (see Table 3, Figure 2).

Covariate paths are reported in Table 4. Sex was positively associated with depression (*β* = 0.09, *p* = 0.008) and with self-harm (*β* = 0.15, *p* = 0.003). Age and left-behind status were not significantly associated with depression, anxiety or self-harm (all *p*s > 0.13). When the model was re-estimated without covariates, the main structural pattern was unchanged (Table 5): peer relationships and father involvement remained linked with self-harm chiefly through depression, and indirect associations via anxiety remained non-significant. Demographic adjustment therefore did not alter the primary conclusions.

In an anxiety-only model that excluded depression (Table 6), the indirect association from peer relationships to self-harm via anxiety was significant (*β* = −0.098, 95% CI [−0.138, −0.059]), whereas the corresponding path for father involvement was not (*β* = −0.012, 95% CI [−0.041, 0.017]). The anxiety pathway was apparent only when depression was omitted from the model. When both mediators were retained, the indirect pathway through depression was the stronger one.

## 4. Discussion

This study employed structural equation modelling to examine associations between peer relationships, father involvement and self-harm among rural Chinese adolescents aged 12–16 years. Main findings indicated that associations between these variables and depression were notably stronger than for anxiety. Neither peer relationships nor father involvement showed a significant direct association with self-harm. Father involvement showed an indirect relationship with self-harm via depression, but not via anxiety. Peer relationships also showed a significant indirect association with self-harm, again, primarily through the peer relationships–depression–self-harm pathway.

We found that depression demonstrated a stronger positive association with self-harm occurrence than anxiety. This finding aligns with Nock and Prinstein’s [37] four-function model, wherein self-harm, as a dysfunctional emotion regulation method, more closely approximates intolerable negative emotions, hopelessness and self-negation. In this sense, depression can be understood as a more critical proximal association channel between social relationship factors and self-harm. This result also echoes Yang and Ma’s [40] symptom network analysis of rural Chinese children, which indicated that depression rather than anxiety serves as the core bridge connecting internalised distress to self-harm. In this study, the indirect associations of father involvement and peer relationships with self-harm ran mainly through depression, which aligns with previous research published by Jiang et al. [53].

In contrast, the anxiety–self-harm link in this study reached only marginal significance, indicating that while anxiety may participate in self-harm risk formation, its role is notably weaker than depression. This does not fully align with the outcomes of Bentley et al.’s [54] review, which suggested significant associations between anxiety disorders and self-harm. This may be because the instrument used, the GAD-7, primarily measures generalised anxiety. Subsequent research using measures of specific forms of anxiety may identify links between anxiety and self-harm. When depression was omitted from the model, the indirect association from peer relationships to self-harm through anxiety was significant, whereas the corresponding association for father involvement was not. With both mediators retained, the pathway through depression was the stronger one.

Among the two types of social protective factors, peer relationships emerged as the more prominent protective factor. Both the total indirect associations and the total associations of peer relationships exceeded those of father involvement. The direct path from peer relationships to self-harm approached zero, with the total association manifesting primarily through the indirect depression pathway. This indicates that the protective associations of peer relationships are likely to operate by buffering internalised distress. Adolescence represents a critical stage when peer importance rises rapidly [55]. For rural adolescents, peer support may play particularly prominent roles in the development of positive emotion regulation strategies and daily stress buffering. By implication, negative peer relationships may also become risk sources. Previous research indicates that peer rejection, bullying and negative peer experiences can increase self-criticism and depression levels, which can in turn increase self-harm rates [28,56].

Compared with peer relationships, the protective association of father involvement was weaker but still significant, similarly manifesting primarily through the depression pathway. Previous research has indicated that father involvement among rural adolescents is significantly associated with lower depression risk [10]. The relatively weaker association may reflect the fact that paternal absences are highly common in rural families. The instrument used in this study primarily assessed companionship and participation behaviours when fathers were present, not in cases of remote involvement. A more specific measure of father involvement may be suitable for use in rural Chinese contexts.

This study has several limitations. First, the design was cross-sectional. Depression and anxiety measures referred to the past two weeks, whereas self-harm was a lifetime yes-or-no report, so it is not possible to tell which came first. The indirect paths do not show that peer relationships or father involvement led to later self-harm through recent symptoms. Second, self-harm was measured with one lifetime yes-or-no question. The question did not record how recent the act was, how often it occurred, how serious it was, which method was used, or whether suicidal intent was present. The findings concern lifetime self-harm on that screen. They do not extend to current or repeated self-harm, or to NSSI as defined in NSSI studies. Third, the father involvement measure mainly reflected companionship and participation when fathers were present; in rural settings where paternal labour migration is common, remote forms of involvement may be under-represented, which may partly explain the comparatively weaker father-involvement associations. Fourth, the models used observed scale totals rather than latent variables, and the primary path model was saturated; the estimates should therefore be read as pathway associations among observed scores, not as a latent structural test. Removing age, sex and left-behind status left the main paths unchanged. Finally, the sample comprised rural adolescents aged 12–16 years from selected schools, so the findings may not generalise to urban adolescents or to other age groups. Longitudinal designs with time-bounded self-harm measures would allow for stronger tests of these pathways.

## 5. Conclusions

Overall, this study further demonstrates that self-harm can be understood within a framework of jointly operating social relationship vulnerabilities and internal emotional distress. Our results indicate that peer relationship quality and father involvement were both negatively associated with self-harm, with depression showing the clearest indirect pathway in the present cross-sectional model. This aligns with previous research on the protective effects of social support in mitigating deliberate self-harm risk [1,4,38]. Depression was the main indirect pathway, so rural schools can start with depressive symptoms.

In prevention work, collaboration across school, family and community is often given less weight, and many programmes still stay in one setting. In mainland China, three-tier collaborative models have been implemented, particularly among children affected by parental HIV infection [57]. These include peer group interventions that enhance self-awareness and interpersonal skills, caregiver training programmes that promote positive parenting practices, and home visits combined with community advocacy to strengthen social support networks and community cohesion [57]. In addition, protective relational factors should be explicitly incorporated into intervention design. At the school level, bullying, friendship quality, and peer influence processes represent critical leverage points for altering developmental trajectories associated with poor mental health outcomes. Given the well-documented links between bullying and internalising symptoms [5,21], effective interventions should extend beyond victims to also address perpetrators and bystanders, with the aim of fostering empathy and reinforcing prosocial norms [58]. Sustained labour migration means that more children in rural China are being raised with their father or with both parents absent from daily life. In rural schools that remain short of services, these results provide a more specific basis for early attention to depressive symptoms and to the lifetime self-harm question, together with peer relationships and father involvement, although this cross-sectional model does not show that a particular programme leads to less self-harm.

## Figures and Tables

**Table 1:** Sample characteristics and descriptive statistics of key variables.

Variable	Category	No Self-Harm (*n* = 432)	Self-Harm (*n* = 156)	Total (*n* = 588)
Demographics				
Sex	Female	224 (51.9%)	97 (62.2%)	321 (54.6%)
	Male	208 (48.1%)	59 (37.8%)	267 (45.4%)
Left-behind status	Non-left-behind	—	—	305 (51.9%)
	Left-behind	—	—	283 (48.1%)
Self-harm rate	Left-behind female	—	—	59/158 (37.3%)
	Non-left-behind female	—	—	45/163 (27.6%)
	Left-behind male	—	—	25/125 (20.0%)
	Non-left-behind male	—	—	27/142 (19.0%)
Key variables: Mean comparisons (*M* ± *SD*)				
Depression (CDI-27)	*M* ± *SD*	12.69 ± 6.97	20.38 ± 8.43	14.69
Anxiety (GAD-7)	*M* ± *SD*	5.11 ± 4.53	8.74 ± 4.73	6.07
Peer relationships	*M* ± *SD*	18.12 ± 6.90	14.63 ± 6.80	17.20
Father involvement	*M* ± *SD*	40.47 ± 20.06	31.31 ± 19.71	37.99

Note. Categorical variables are *n* (%); continuous variables are *M* ± *SD*. Totals refer to the full adolescent sample (*N* = 588) and are unweighted. Self-harm rates by sex and left-behind status are *n*/*N* (%), where *N* is the number of participants in that subgroup. Left-behind status indicates that the father or both parents had migrated for work. Group differences on all continuous variables (no self-harm vs. self-harm) were significant (*p*s < 0.001).

**Table 2:** Standardised direct paths identified by the structural equation modelling.

Path	*β*	SE	95% CI	*p*	
Peer relationships → Depression	−0.45	0.03	[−0.52, −0.39]	<0.001	***
Father involvement → Depression	−0.16	0.04	[−0.24, −0.08]	<0.001	***
Peer relationships → Anxiety	−0.29	0.04	[−0.37, −0.21]	<0.001	***
Father involvement → Anxiety	−0.03	0.04	[−0.12, 0.05]	0.42	
Depression → Self-harm	0.37	0.07	[0.24, 0.50]	<0.001	***
Anxiety → Self-harm	0.12	0.07	[−0.01, 0.25]	0.06	
Peer relationships → Self-harm	−0.03	0.06	[−0.15, 0.10]	0.66	
Father involvement → Self-harm	−0.10	0.06	[−0.21, 0.01]	0.09	

Note. *** *p* < 0.001. Age, sex and left-behind status were included in all three outcome equations (see Table 4). Re-estimation without these covariates left the main structural pattern unchanged (see Table 5). The model is saturated (df = 0); global fit indices are not interpreted. An anxiety-only mediation check is reported in Table 6.

**Table 3:** Indirect and total associations from the main model.

Association	*β*	SE	95% CI	*p*
Peer relationships → Depression → Self-harm	−0.17	0.03	[−0.23, −0.10]	<0.001
Peer relationships → Anxiety → Self-harm	−0.03	0.02	[−0.07, 0.00]	0.07
Father involvement → Depression → Self-harm	−0.06	0.02	[−0.10, −0.02]	<0.01
Father involvement → Anxiety → Self-harm	0.00	0.01	[−0.02, 0.01]	0.46
Peer relationships: total indirect effect	−0.20	0.03	[−0.26, −0.15]	<0.001
Father involvement: total indirect effect	−0.06	0.02	[−0.11, −0.02]	<0.01
Peer relationships: total effect	−0.23	0.06	[−0.34, −0.12]	<0.001
Father involvement: total effect	−0.16	0.06	[−0.28, −0.05]	0.01

Note. Standardised indirect and total associations estimated using the product-of-coefficients method. 95% CI derived using the delta method. R^2^ (depression) = 0.31; R^2^ (anxiety) = 0.11; R^2^ (self-harm) = 0.31.

**Table 4:** Standardised covariate paths in the primary model.

Outcome	Covariate	Coding	*β*	SE	95% CI	*p*
Depression	Age	Years	−0.040	0.034	[−0.106, 0.027]	0.241
Depression	Sex	0 = male, 1 = female	0.092	0.035	[0.024, 0.161]	0.008
Depression	Left-behind	0 = no, 1 = yes	0.030	0.035	[−0.038, 0.099]	0.390
Anxiety	Age	Years	−0.061	0.041	[−0.141, 0.019]	0.133
Anxiety	Sex	0 = male, 1 = female	0.045	0.040	[−0.032, 0.123]	0.254
Anxiety	Left-behind	0 = no, 1 = yes	0.046	0.040	[−0.033, 0.124]	0.251
Self-harm	Age	Years	−0.021	0.050	[−0.119, 0.076]	0.668
Self-harm	Sex	0 = male, 1 = female	0.148	0.051	[0.049, 0.247]	0.003
Self-harm	Left-behind	0 = no, 1 = yes	−0.005	0.052	[−0.107, 0.097]	0.926

Note. Estimates are from the covariate-adjusted WLSMV/probit path model.

**Table 5:** Key structural paths with versus without covariates.

Path	With Covariates *β* (*p*)	Without Covariates *β* (*p*)
Peer → Depression	−0.455 (<0.001)	−0.455 (<0.001)
Father → Depression	−0.161 (<0.001)	−0.172 (<0.001)
Peer → Anxiety	−0.288 (<0.001)	−0.294 (<0.001)
Father → Anxiety	−0.035 (0.416)	−0.041 (0.337)
Depression → Self-harm	0.369 (<0.001)	0.399 (<0.001)
Anxiety → Self-harm	0.121 (0.064)	0.114 (0.084)
Peer → Self-harm (direct)	−0.027 (0.663)	−0.005 (0.939)
Father → Self-harm (direct)	−0.098 (0.087)	−0.105 (0.056)
Peer → Depression → Self-harm	−0.168 (sig)	−0.182 (sig)
Father → Depression → Self-harm	−0.060 (sig)	−0.069 (sig)
Peer → Anxiety → Self-harm	−0.035 (ns)	−0.034 (ns)
Father → Anxiety → Self-harm	−0.004 (ns)	−0.005 (ns)

Note. “sig” = 95% CI excludes 0; “ns” = 95% CI includes 0. Primary conclusions are unchanged after removing covariates.

**Table 6:** Indirect associations from the anxiety-only model.

Effect	*β*	95% CI
Peer → Anxiety → Self-harm	−0.098	[−0.138, −0.059]
Father → Anxiety → Self-harm	−0.012	[−0.041, 0.017]

## Data Availability

The data presented in this study are openly available in the Science Data Bank at https://doi.org/10.57760/sciencedb.j00001.00464.

## Abbreviations

The following abbreviations are used in this manuscript:

CDI-27	Children’s Depression Inventory–27 items
GAD-7	Generalized Anxiety Disorder–7
FIQ	Father Involvement Questionnaire
PR	Peer Relationships Scale
NSSI	Non-suicidal self-injury
SEM	Structural equation modelling
WLSMV	Weighted least squares mean and variance-adjusted
CI	Confidence interval
SE	Standard error
*SD*	Standard deviation
*M*	Mean
*N*/*n*	Sample size
df	Degrees of freedom
*β*	Standardised path coefficient
R^2^	Coefficient of determination
